# A Large-Scale Genomic Association Analysis Identifies the Candidate Genes Regulating Salt Tolerance in Cucumber (*Cucumis sativus* L.) Seedlings

**DOI:** 10.3390/ijms23158260

**Published:** 2022-07-27

**Authors:** Dongrang Liu, Shaoyun Dong, Han Miao, Xiaoping Liu, Caixia Li, Jianan Han, Shengping Zhang, Xingfang Gu

**Affiliations:** Institute of Vegetables and Flowers, Chinese Academy of Agricultural Sciences, Beijing 100081, China; 82101192231@caas.cn (D.L.); dongshaoyun@caas.cn (S.D.); miaohan@caas.cn (H.M.); liuxiaoping@caas.cn (X.L.); 82101211129@caas.cn (C.L.); 82101191124@caas.cn (J.H.)

**Keywords:** cucumber seedlings, salt stress, GWAS, candidate genes

## Abstract

Salt stress seriously restricts plant growth and development, affects yield and quality, and thus becomes an urgent problem to be solved in cucumber stress resistance breeding. Mining salt tolerance genes and exploring the molecular mechanism of salt tolerance could accelerate the breeding of cucumber germplasm with excellent salt stress tolerance. In this study, 220 cucumber core accessions were used for Genome-Wide Association Studies (GWAS) and the identification of salt tolerance genes. The salinity injury index that was collected in two years showed significant differences among the core germplasm. A total of seven loci that were associated with salt tolerance in cucumber seedlings were repeatedly detected, which were located on Chr.2 (*gST2.1*), Chr.3 (*gST3.1* and *gST3.2*), Chr.4 (*gST4.1* and *gST4.2*), Chr.5 (*gST5.1*), and Chr.6 (*gST6.1*). Within these loci, 62 genes were analyzed, and 5 candidate genes (*CsaV3_2G035120*, *CsaV3_3G023710*, *CsaV3_4G033150*, *CsaV3_5G023530*, and *CsaV3_6G009810*) were predicted via the functional annotation of *Arabidopsis* homologous genes, haplotype of extreme salt-tolerant accessions, and qRT-PCR. These results provide a guide for further research on salt tolerance genes and molecular mechanisms of cucumber seedlings.

## 1. Introduction

Cucumber (*Cucumis sativus* L.) is one of the most important vegetables worldwide, and according to the FAOSTAT (https://www.fao.org/faostat/zh/#home (accessed on 17 January 2022)), the global total grown area and production of cucumber was 2.25 million hectares and 90.35 million tons, respectively, in 2020. However, cucumber is very sensitive to salt stress, which is one of the important limiting factors in cucumber cultivation [1,2].

In general, salt stress restricts plant physiological activity through osmotic and toxic effects, depending on the salt concentration [3]. Previous studies have shown that plants have evolved multiple complex mechanisms to adapt to salt stress in environment, including antioxidant systems, osmotic regulation, transcriptional regulation, and signal transduction, etc. [4,5]. Salt stress will cause significant damage to cucumber seedlings, which is mainly manifested in obvious symptoms of chlorosis and the decline of various growth indicators of the plant and root system, and the damage is significantly increased with the increase of salt concentration and treatment time [6,7]. Therefore, mining genes that are related to plant salt tolerance is an important and complex process. Researches have shown that cucumber salt tolerance is a quantitative trait that is controlled by multiple genes and is easily affected by environmental factors [7,8]. Liu, et al. [7] evaluated the salt tolerance of a RILs population in cucumber seedlings and detected a major QTL on chromosome 6 using salt injury index. As this is currently the only reported QTL controlling the salt tolerance of cucumber seedlings, it is urgent to identify more loci and genes to elucidate the mechanism of cucumber salt tolerance.

Recently, linkage mapping and genome-wide association studies have been widely used in mining genes that are related to vegetable phenotypic variation [9]. Hou, et al. [10] conducted GWAS analysis on 212 diverse melon (*Cucumis melo* L.) accession seedlings using chilling injury index and identified 11 low-temperature tolerance genes. Khanzada, et al. [11] identified the phenotypes of 228 *Brassica* accession seedlings under drought stress and 85 drought tolerance genes were identified by GWAS analysis in rapeseed (*Brassica napus*). Zhang, et al. [12] used GWAS analysis of 83 non-heading Chinese cabbage accessions to identify 17 loci that were associated with turnip mosaic virus (TuMV) resistance. In cucumber, a large number of loci and genes that are related to cucumber growth phenotypes and resistance traits have been unearthed through GWAS analysis. Wei, et al. [13] used the heat injury index of 86 cucumber core accessions to conduct GWAS analysis and detected seven GWAS loci (*gHII4.1*, *gHII4.2*, *gHII5.1*, *gHII5.2*, *gHII6.1*, *gHII6.2,* and *gHII7.1*). Wang, et al. [14] repeatedly detected four GWAS loci (*gLTS1.1*, *gLTS3.1*, *gLTS4.1*, and *gLTS5.1*) by using the chilling injury index of 172 cucumber core accessions seedlings. Lee, et al. [15] conducted GWAS analysis of cucumber powdery mildew resistance using 264 accessions from all over the world and predicted two novel candidate genes (*Csa5G453160* and *Csa5G471070*). Liu, et al. [16,17] used the core germplasm to predict five downy mildew-related candidate genes (*Csa1G575030*, *Csa2G060360*, *Csa4G064680*, *Csa5G606470*, and *Csa7G004020*) and eight candidate genes that were associated with powdery mildew (*Csa2G030010*, *Csa2G030020*, *Csa2G030030*, *Csa3G414050*, *Csa4G022350*, *Csa5G48880*, *Csa5G603950*, and *Csa5G603960*), respectively. However, no study on the investigation of cucumber salt tolerance via GWAS has been reported.

Here, GWAS was performed using the salt injury index of 220 cucumber core accessions, the loci that were repeatedly detected in two years, that were further investigated, and candidate genes were mined through haplotype and qRT-PCR analyses. These genes could lay the foundation for the elaboration of cucumber salt stress response mechanism and the molecular improvement of excellent salt-tolerant cucumber varieties.

## 2. Results

### 2.1. Genetic Diversity of Salt Tolerance in Cucumber Germplasm

**Figure 1:**
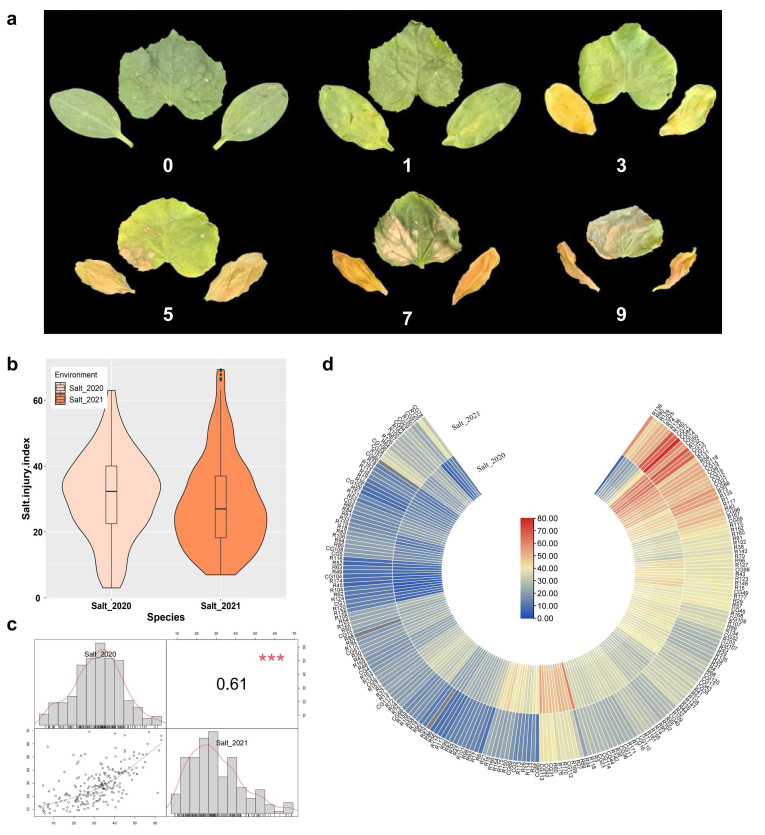
Phenotypic characterization of salt tolerance in 14 day old cucumber seedlings that were treated with 200 mmol·L^−1^ NaCl for seven days. A total of three replicates were set for each experiment, with a total of 15 replicates per accession. (**a**) Salt injury of cucumber seedlings was divided into 6 grades. 0, the cotyledons and the first true leaf show no symptoms of salt injury; 1, the cotyledons are slightly yellowed; 3, the cotyledons and the first true leaf are significantly yellowed; 5, the cotyledons are yellowed and wilted, and the first true leaf is significantly yellowed and wilted; 7, the cotyledons are dehydrated, the large areas of first true leaf are yellowed and wilted; 9, both the cotyledons and the first true leaf are yellowed and dehydrated. (**b**) Violin plot depicting the phenotypic distribution of salt injury index in the two treatments. (**c**) Frequency distribution and Pearson rank correlations of the mean salt injury index in two experiments. *** indicate significance at *p* < 0.001. (**d**) Heatmap depicting the salt injury index in two treatments.

The 220 accessions were evaluated for salt tolerance two times, in June 2020 (Salt_2020) and May 2021 (Salt_2021). A total of three replicates were set for each experiment, each of which had 5 plants. At 14 days old, the seedlings were exposed to salt stress by 200 mmol·L^−1^ NaCl. After 7 days of salt treatment, the salt injury was divided into six scales, based on the chlorosis and wilting degree of the first true leaf and cotyledons (Figure 1a). For each accession, the salt injury index of each experiment was calculated by the formula on the basis of the six scales and shown in Table S2. The phenotypic data of the two treatments showed that the salt injury index of different accessions was significantly different. The average salt injury index was 31.18 and 28.80, with the coefficient of variation of 41.35% and 47.84%, respectively (Table 1). As shown in Table 1 and Figure 1c, the normal distribution test (*p* > 0.05) and the violin plot showed that the salt injury index follow the normal distribution pattern, and the salt injury index correlation among the two experiments was significant (Figure 1c,d).

**Figure 2:**
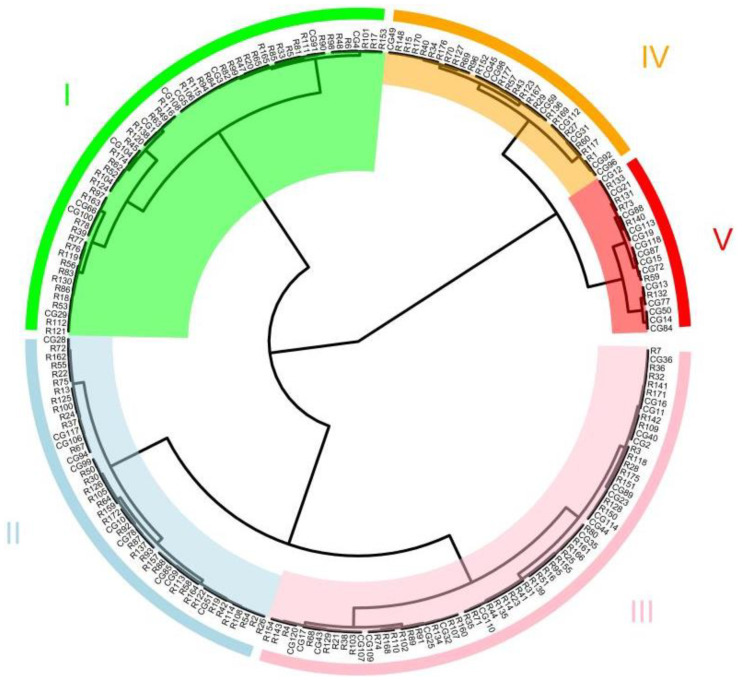
Cluster analysis of salt tolerance of core accession seedlings based on salt injury index. The I, II, III, IV, and V indicates Group I, Group II, Group III, Group IV, and Group V, respectively.

**Figure 3:**
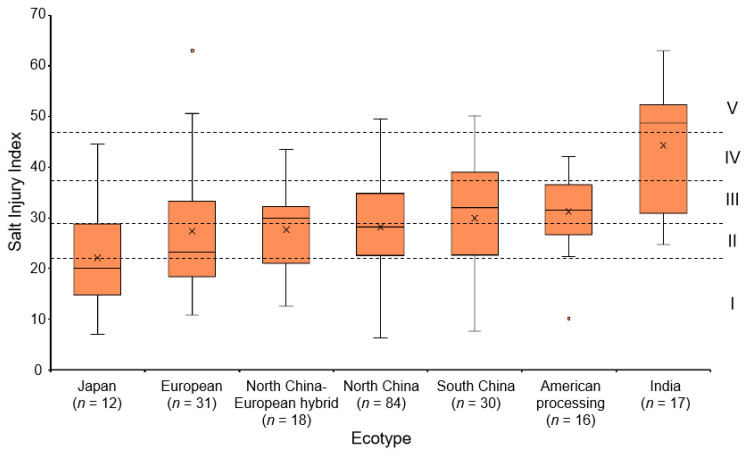
The box plot depicting the salt injury index of different ecotypes. The I, II, III, IV, and V indicates Group I, Group II, Group III, Group IV, and Group V, respectively. The dot represents the outlier. The cross represents the mean.

According to difference in the salt injury index, 220 accessions were divided into 5 groups (I, II, III, IV, and V) by the Ward method (Figure 2). Group Ⅰ had 58 highly salt-tolerant accessions, Group Ⅱ had 45 salt-tolerant accessions, Group Ⅲ had 66 intermediate salt-tolerant accessions, Group Ⅳ had 31 salt-sensitive accessions, and Group Ⅴ had 20 highly salt-sensitive accessions (Table S3). The results of cluster analysis were further analyzed in combination with the ecotype of the germplasm. The 220 core accessions included 1 European-Asia ecotype, 5 American slicing ecotypes, 6 Xishuangbanna ecotypes, 12 Japan ecotypes, 16 American processing ecotypes, 17 India ecotypes, 18 North China-European hybrid ecotypes, 30 South China ecotypes, 31 European ecotypes, and 84 North China ecotypes. It was difficult to conclude the salt tolerance of the Xishuangbanna ecotype, American slicing ecotype, and the European-Asia ecotype, since only several lines were included. As such, we analyzed the ecotypes which contained more than 10 accessions. The results showed that most of the Japan ecotypes showed high salt-tolerance; the European, North China-European hybrid and North China ecotypes exhibited intermediate salt-tolerance; while the Indian ecotypes were more salt-sensitive (Figure 3).

### 2.2. Genome-Associated Analysis of Salt Tolerance in Seedlings

**Figure 4:**
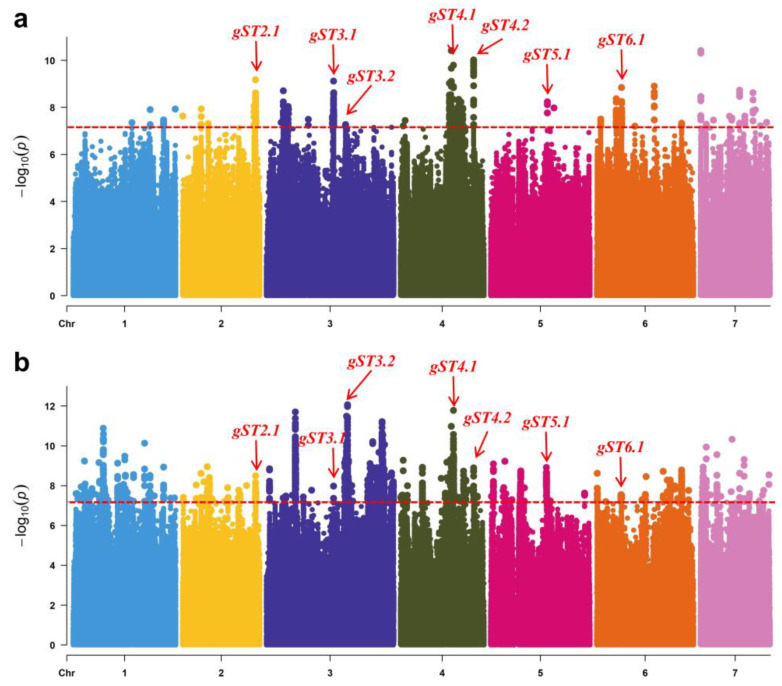
Genome-wide association analysis (GWAS) Manhattan plots of salt tolerance in two experiments: (**a**) Salt_2020 and (**b**) Salt_2021. The different colors represent the different chromosomes. The red dashed line represents the significance threshold. The red arrow indicates the position of the strong peaks that were repeatedly detected in the two experiments.

Using the salt injury index data of the two experiments in 2020 and 2021, genome-wide association analysis (GWAS) was performed and 7 genetic loci (*gST2.1*, *gST3.1*, *gST3.2*, *gST4.1*, *gST4.2*, *gST5.1,* and *gST6.1*) that were repeatedly detected. These 7 loci were distributed over five chromosomes (Figure 4), which were considered as novel loci for salt tolerance in cucumber seedlings.

### 2.3. Candidate Gene Analysis for the Novel Loci

In order to identify the potential candidate genes in these loci, the 100-kb region around each peak of SNPs was used for further analysis, and genes in each locus are shown in Table S4. To identify the candidate genes, functional annotation of the orthologs in *Arabidopsis*, haplotype of extreme salt-tolerant accessions, and qRT-PCR were analyzed. According to the results of the clustering analysis, Group Ⅰ (58 highly salt-tolerant accessions) and Group Ⅴ (20 highly salt-sensitive accessions) were used for haplotype analysis. Based on the phenotypic data and ecotype of accessions, salt-tolerant accessions (R45, R63, R78, and CG104) and salt-sensitive accessions (R59, R132, CG13, and CG14) were used for expression analysis. At 14 days old, the seedlings were exposed to salt stress by 200 mmol·L^−1^ NaCl, with a total of 4 time points, namely 0 h, 10 h, 48 h, and 96 h post-salt treatment. The first true leaf of each seedling was harvested as the sample for qRT-PCR. Through the above analysis, we preliminarily predicted five candidate genes that were related to salt tolerance in *gST2.1*, *gST3.1*, *gST4.2*, *gST5.1*, and *gST6.1* (Table S5).

**Figure 5:**
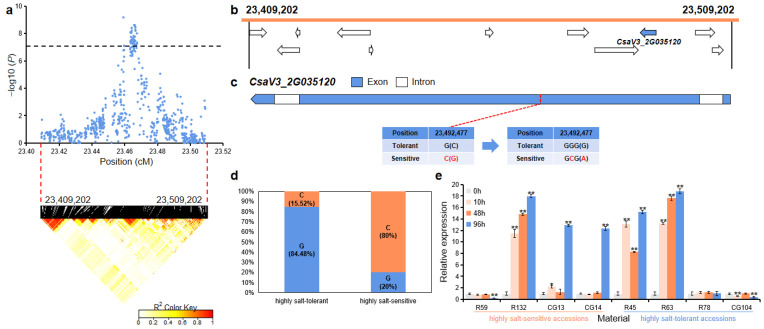
Identification of the candidate gene that was related to salt tolerance for the locus *gST2.1*. (**a**) Manhattan plot and LD heatmap of *gST2.1*. The black dashed line represents the significance threshold and the red dashed lines represent the candidate region (100 kb). (**b**) A total of 11 functional annotated genes were predicted in the *gST2.1* candidate region. The blue arrow indicates that the functional annotation of the gene which is related to salt tolerance. (**c**) Gene structure of *CsaV3_2G035120*. Blue rectangles and white rectangles indicate exons and introns, respectively. (**d**) Orange and blue blocks represent the proportion of C and G haplotype in highly salt-tolerant or highly salt-sensitive accessions, respectively. (**e**) qRT-PCR was used to detect the expression changes of *CsaV3_2G035120* in highly salt-sensitive accessions (R59, R132, CG13 and CG14) and highly salt-tolerant accessions (R45, R63, R78 and CG104) after salt treatment (0 h, 10 h, 48 h and 96 h). Significant difference (* *p* < 0.05, ** *p* < 0.01).

For *gST2.1*, the 100 kb (23,409,202~23,509,202 bp) region on Chr.2 was analyzed using pairwise LD correlations (Figure 5a). According to CuGenDB, 11 annotated genes are located in this region, and there was one gene (*CsaV3_2G035120*) that was related to salt stress (Figure 5b and Table S5). *CsaV3_2G035120* (encoding an isocitrate lyase) had a SNP in the second exon region that led to the mutation of glycine to alanine (Figure 5c). In the highly salt-tolerant and highly salt-sensitive accessions, 49 out of the 58 highly salt-tolerant accessions (84.48%) carried the G haplotype, while 16 out of the 20 highly salt-sensitive (80%) accessions had the alternate C haplotype (Figure 5d). qRT-PCR analysis revealed that the relative expression level of this gene was significantly up-regulated in highly salt-tolerant accessions (R45 and R63) and highly salt-sensitive accessions (R132, CG13, and CG14) after salt treatment (Figure 5e).

**Figure 6:**
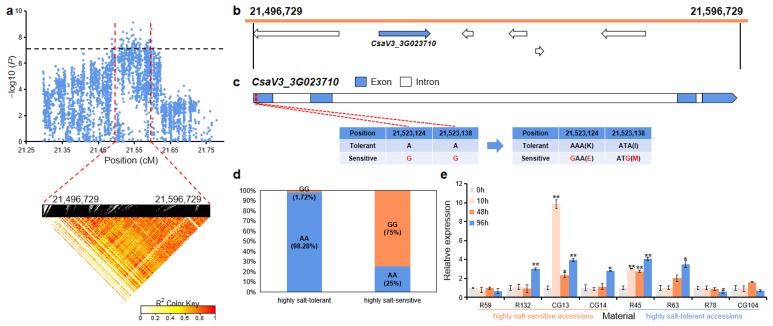
Identification of the candidate gene that was related to salt tolerance for the locus *gST3.1*. (**a**) Manhattan plot and LD heatmap of *gST3.1*. The black dashed line represents the significance threshold and the red dashed lines represent the candidate region (100 kb). (**b**) A total of six functional annotated genes were predicted in the *gST3.1* candidate region. The blue arrow indicates that the functional annotation of the gene which is related to salt tolerance. (**c**) Gene structure of *CsaV3_3G023710*. Blue rectangles and white rectangles indicate exons and introns, respectively. (**d**) Orange and blue blocks represent the proportion of GG and AA haplotype in the highly salt-tolerant or highly salt-sensitive accessions, respectively. (**e**) qRT-PCR was used to detect the expression changes of *CsaV3_3G023710* in the highly salt-sensitive accessions (R59, R132, CG13, and CG14) and highly salt-tolerant accessions (R45, R63, R78, and CG104) after salt treatment (0 h, 10 h, 48 h, and 96 h). Significant difference (* *p* < 0.05, ** *p* < 0.01).

For *gST3.1*, the 100 kb (21,496,729~21,596,729 bp) region on Chr.3 was analyzed using pairwise LD correlations (Figure 6a). According to CuGenDB, six annotated genes are located in this region, and there was one gene (*CsaV3_3G023710*) that was related to salt stress (Figure 6b and Table S5). *CsaV3_3G023710* (encoding an ankyrin repeat family protein) had two SNPs in the first exon region that led to the mutation of lysine to glutamate and isoleucine to methionine (Figure 6c). In the highly salt-tolerant and highly salt-sensitive accessions, 57 out of the 58 highly salt-tolerant accessions (98.28%) carried the AA haplotype, while 15 out of the 20 highly salt-sensitive (75%) accessions had the alternate GG haplotype (Figure 6d). qRT-PCR analysis revealed that the relative expression level of this gene was significantly up-regulated in the highly salt-tolerant accessions (R45 and R63) and the highly salt-sensitive accessions (R132, CG13, and CG14) after salt treatment (Figure 6e).

For *gST3.2* and *gST4.1*, we found two genes (*CsaV3_3G030250* and *CsaV3_4G027680*) that were related to salt stress according to CuGenDB. There were nine SNPs on *CsaV3_3G030250* and one SNP on *CsaV3_4G027680*, which were located in the CDS (coding sequence) region that led to amino acid variation (Figures S1c and S2c). But according to the haplotype analysis, all of these SNPs did not have any differential change between the highly salt-tolerant and highly salt-sensitive accessions (Figures S1d and S2d). As such, the candidate genes in loci *gST3.2* and *gST4.1* had not been predicted.

**Figure 7:**
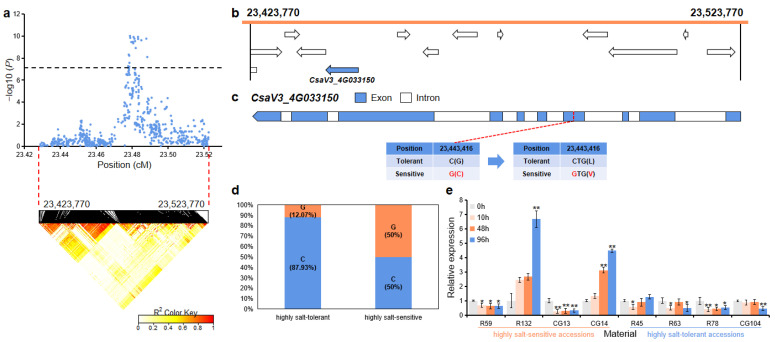
Identification of the candidate gene that was related to salt tolerance for the locus *gST4.2*. (**a**) Manhattan plot and LD heatmap of *gST4.2*. The black dashed line represents the significance threshold and the red dashed lines represent the candidate region (100 kb). (**b**) A total of 13 functional annotated genes were predicted in the *gST4.2* candidate region. The blue arrow indicates that the functional annotation of the gene which is related to salt tolerance. (**c**) Gene structure of *CsaV3_4G033150*. Blue rectangles and white rectangles indicate exons and introns, respectively. (**d**) Orange and blue blocks represent the proportion of G and C haplotypes in the highly salt-tolerant or highly salt-sensitive accessions, respectively. (**e**) qRT-PCR was used to detect the expression changes of *CsaV3_4G033150* in the highly salt-sensitive accessions (R59, R132, CG13, and CG14) and highly salt-tolerant accessions (R45, R63, R78, and CG104) after salt treatment (0 h, 10 h, 48 h, and 96 h). Significant difference (* *p* < 0.05, ** *p* < 0.01).

For *gST4.2*, the 100 kb (23,423,770~23,523,770 bp) region on Chr.4 was analyzed using pairwise LD correlations (Figure 7a). According to CuGenDB, 13 annotated genes are located in this region, and there was one gene (*CsaV3_4G033150*) that was related to salt stress (Figure 7b and Table S5). *CsaV3_4G033150* (encoding a phosphoenolpyruvate carboxylase protein) had one SNP in the fourth exon region that led to the mutation of leucine to valine (Figure 7c). In the highly salt-tolerant and highly salt-sensitive accessions, 51 out of the 58 highly salt-tolerant accessions (87.93%) carried the C haplotype, while 10 out of the 20 highly salt-sensitive (50%) accessions had the alternate G haplotype (Figure 7d). qRT-PCR analysis revealed that after salt treatment, the relative expression level of this gene was significantly down-regulated in the highly salt-tolerant accessions (R45, R63, R78, and CG104), while was significantly up-regulated in the highly salt-sensitive accessions (R132 and CG14) (Figure 7e).

**Figure 8:**
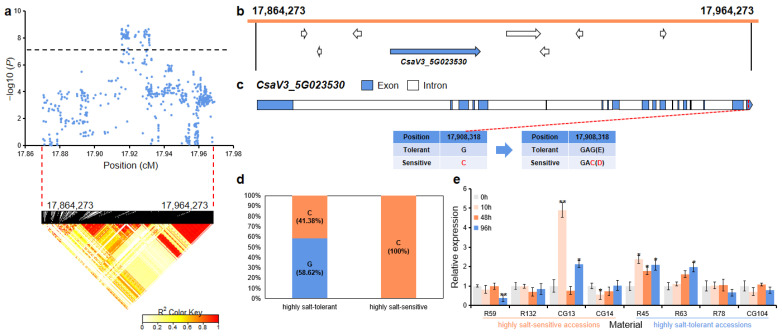
Identification of the candidate gene that was related to salt tolerance for the locus *gST5.1*. (**a**) Manhattan plot and LD heatmap of *gST5.1*. The black dashed line represents the significance threshold and the red dashed lines represent the candidate region (100 kb). (**b**) A total of eight functional annotated genes were predicted in the *gST5.1* candidate region. The blue arrow indicates that the functional annotation of the gene which is related to salt tolerance. (**c**) The gene structure of *CsaV3_5G023530*. Blue rectangles and white rectangles indicate exons and introns, respectively. (**d**) Orange and blue blocks represent the proportion of C and G haplotypes in the highly salt-tolerant or highly salt-sensitive accessions, respectively. (**e**) qRT-PCR was used to detect the expression changes of *CsaV3_5G023530* in the highly salt-sensitive accessions (R59, R132, CG13, and CG14) and highly salt-tolerant accessions (R45, R63, R78, and CG104) after salt treatment (0 h, 10 h, 48 h, and 96 h). Significant difference (* *p* < 0.05, ** *p* < 0.01).

For *gST5.1*, the 100 kb (17,864,273~17,964,273 bp) region on Chr.5 was analyzed using pairwise LD correlations (Figure 8a). According to CuGenDB, eight annotated genes are located in this region, and there was one gene (*CsaV3_5G023530*) that was related to salt stress (Figure 8b and Table S5). *CsaV3_5G023530* (encoding a PHD finger protein) had one SNP in the 19th exon region that led to the mutation of glutamate to aspartate (Figure 8c). In the highly salt-tolerant and highly salt-sensitive accessions, 24 out of the 58 highly salt-tolerant accessions (58.62%) carried the G haplotype, while all highly salt-sensitive (100%) accessions had the alternate C haplotype (Figure 8d). qRT-PCR analysis revealed that after salt treatment, the relative expression level of this gene was significantly up-regulated in the highly salt-tolerant accessions (R45 and R63), while was significantly down-regulated in the highly salt-sensitive accessions (R59 and CG14) (Figure 8e).

**Figure 9:**
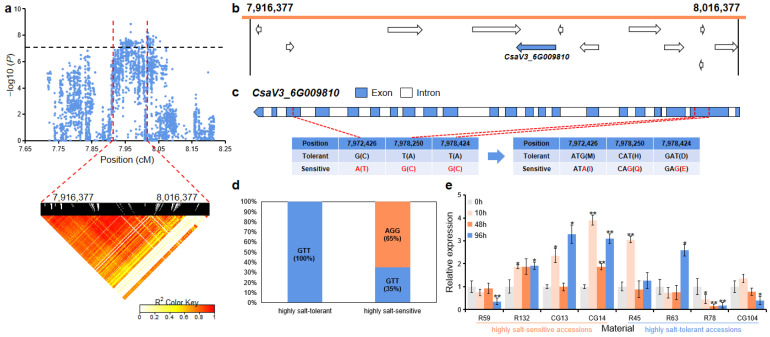
Identification of the candidate gene that as related to salt tolerance for the locus *gST6.1*. (**a**) Manhattan plot and LD heatmap of *gST5.1*. The black dashed line represents the significance threshold and the red dashed lines represent the candidate region (100 kb). (**b**) A total of 12 functional annotated genes were predicted in the *gST6.1* candidate region. The blue arrow indicates that the functional annotation of the gene which is related to salt tolerance. (**c**) Gene structure of *CsaV3_6G009810*. Blue rectangles and white rectangles indicate exons and introns, respectively. (**d**) Orange and blue blocks represent the proportion of AGG and GTT haplotype in the highly salt-tolerant or highly salt-sensitive accessions, respectively. (**e**) qRT-PCR was used to detect the expression changes of *CsaV3_6G009810* in the highly salt-sensitive accessions (R59, R132, CG13, and CG14) and highly salt-tolerant accessions (R45, R63, R78, and CG104) after salt treatment (0 h, 10 h, 48 h, and 96 h). Significant difference (* *p* < 0.05, ** *p* < 0.01).

For *gST6.1*, the 100 kb (7,916,377~8,016,377 bp) region on Chr.6 was analyzed using pairwise LD correlations (Figure 9a). According to CuGenDB, 12 annotated genes are located in this region, and there was one gene (*CsaV3_6G009810*) that was related to salt stress (Figure 9b and Table S5). *CsaV3_6G009810* (encoding a pesticidal crystal cry8Ba protein) had three SNPs in the CDS (coding sequence) region that led to the mutation of aspartate to glutamate, histidine to glutamine, and methionine to isoleucine (Figure 9c). In the highly salt-tolerant and highly salt-sensitive accessions, all the highly salt-tolerant accessions (100%) carried the GTT haplotype, while 13 out of the 20 highly salt-sensitive (65%) accessions had the alternate AGG haplotype (Figure 9d). qRT-PCR analysis revealed that after salt treatment, the relative expression level of this gene was significantly up-regulated in the highly salt-tolerant accessions (R45 and R63) and highly salt-sensitive accessions (R132, CG13, and CG14), while was significantly down-regulated in the highly salt-tolerant accessions (R78 and CG104) (Figure 9e).

## 3. Discussion

The acquisition of phenotypic data is the basis of GWAS. At present, there are many studies on the identification of salt tolerance phenotypic data of cucumber, involving morphological indicators, physiological and biochemical indicators, and cytological indicators [18]. We obtained effective phenotypic data by using the salt injury index classification survey [7]. It can efficiently obtain germplasm salt injury index data that can be used for subsequent analysis. As such, we identified the salt tolerance of different accessions by referring to this method (Figure 1a). Meanwhile, we conducted the experiments in two different environments (Shouguang in 2020 and Beijing in 2021) to ensure the accuracy of the results. The results showed that the two phenotypic data in this study have significant correlations, demonstrating the reliability of the data and the applicability of this method (Table 1 and Figure 1b).

The stress resistance of accessions in different ecotype groups was different. Cao, et al. [19] believed that the salt tolerance of South China ecotype cucumber germplasm was generally better than that of North China cucumber germplasm at the germination stage. Wei, et al. [13] identified the heat injury index of 86 cucumber core accession seedlings and found that the Indian ecotype and Xishuangbanna ecotype accessions were mostly highly heat-tolerant. Wang, et al. [14] used the chilling injury index of 87 cucumber core accession seedlings as an indicator and found that most of the North China ecotype and Japan ecotype accessions were tolerant to low temperature, while most of the European ecotype and India ecotype accessions were sensitive to low temperature. In this study, 220 core accessions were used to identify the salt tolerance of seedlings and we found that Japan ecotype and Indian ecotype accessions were generally highly salt-tolerant and highly salt-sensitive, respectively (Figure 3).

In recent years, great progress has been made in revealing the key genes of plant salt tolerance through GWAS in a variety of crops. Baxter, et al. [20] used 337 *Arabidopsis* accessions to conduct GWAS analysis of Na^+^ accumulation in leaves under salt stress and identified the key locus of *Arabidopsis* salt tolerance *AtHKT1;1* via combined genetic complementation and gene expression studies. Patishtan, et al. [21] used 306 rice accessions as materials and used Na^+^ and K^+^ content in the tissues as indictors for GWAS analysis and predicted 70 candidate genes. Do, et al. [22] detected a GWAS locus that was associated with four traits, including leaf scorch degree, chlorophyll content ratio, leaf sodium content, and leaf chlorine content using 305 soybean accessions. Oyiga, et al. [23] used 150 wheat accessions to conduct GWAS analysis with Na^+^ and K^+^ contents in leaves and predicted 22 candidate genes for salt tolerance with combined gene expression analysis. Sun, et al. [24] measured the relative survival rate and salt tolerance of 713 cotton accessions under salt stress and predicted six candidate genes by combining GWAS analysis and expression analysis. In this study, *gST2.1*, *gST3.1*, *gST3.2*, *gST4.1*, *gST4.2*, *gST5.1,* and *gST6.1* were new salt tolerance loci in cucumber through the identification of salt tolerance of core germplasm and GWAS analysis, which was also the first application of GWAS analysis in mining salt tolerance genes in cucumber (Figure 4 and Table S4).

The mechanism of cucumber response to salt stress is a complex and dynamic process. Through homologous gene cloning and expression analysis, it was found that the main mechanisms of salt stress regulation in *Arabidopsis* also play an important role in cucumber, such as SOS (salt-overly-sensitive) and ABA [18]. Currently, a QTL locus that is associated with salt tolerance in cucumber has been reported [7]. In addition, a large number of gene families that might be related to salt tolerance of cucumber have been identified in recent years, such as CDPK [25], SOD [26], AQP [27], bHLH [28], WRKY [29], GRX [30], and CAMTA [31]. In this study, five candidate genes that were associated with salt stress were predicted from these seven loci (Table S5). *CsaV3_2G035120* encodes a glyoxylate cycle enzyme isocitrate lyase (ICL) that is involved in salt tolerance. Transcriptome analysis showed that rice isocitrate lyase gene (*OsICL*) was induced by salt stress, and the overexpression of rice calmodulin gene (*OsCAM1-1*) enhanced *OsICL* expression by salt stress [32]. The overexpression of *OsICL* in *Arabidopsis AtICL* knockout mutant by Yuenyong, et al. [33] further supports the role of ICL in plant salt tolerance through the glyoxylate cycle. Our study found the expression of *CsaV3_2G035120* was also significantly up-regulated by salt stress in cucumber core accessions (R45, R63, R132, CG13, and CG14) (Figure 5e).

*CsaV3_3G023710* encodes an ankyrin repeat family protein and the homologous gene in *Arabidopsis* is *ITN1* (*AT3G12360*). *ITN1* encodes a protein with an ankyrin motif and transmembrane domains that is involved in salt tolerance and may act through abscisic acid (ABA) signaling pathways and promote reactive oxygen species (ROS) production in *Arabidopsis* [34]. The expression of *CsaV3_3G023710* was also significantly up-regulated by salt stress in highly salt-sensitive cucumber core accessions (R132, CG13, and CG14) (Figure 6e).

*CsaV3_4G033150* encodes *Arabidopsis* phosphoenolpyruvate carboxylase (PEPC) proteins. Sanchez, et al. [35] found that *PEPC* genes show differential expression in *Arabidopsis* organs in response to salt and drought. Zhong, et al. [36] found the expression of the *PEPC* gene in cucumber leaves increased under NaCl stress. We also found that *CsaV3_4G033150* expression was significantly up-regulated in two highly salt-sensitive accessions (R132 and CG14), while significantly down-regulated in all the highly salt-tolerant accessions (Figure 7e).

*CsaV3_5G023530* encodes a plant homologous domain (PHD) finger family protein. PHD finger proteins are a family of important zinc finger transcription factors that are responsible for regulating the transcription and chromatin state and responding to various stresses, and this family of genes has been reported to be associated with salt tolerance in many plants, such as *Arabidopsis thaliana* [37], *Moso bamboo* [38], *Brassica rapa* [39], and *Solanum tuberosum* [40]. Zheng, et al. [41] found that the expression of *CsaV3_5G023530* homologous gene *PRE2* (*AT1G77800*) in *Arabidopsis* was significantly increased under salt treatment, which may be involved in ABA and salt responses in *Arabidopsis*. We also found that the expression level of *CsaV3_5G023530* changed significantly in several cucumber accessions under salt stress, especially in the highly salt-sensitive accession CG13, which showed an extremely significant increase (Figure 8e).

*CsaV3_6G009810* is predicted to encode a pesticidal crystal cry8Ba protein in CuGenDB, but more information in NCBI shows that it encodes a peptide of unknown function with no similarity to any known sequence to date. *AtRASD1* (*AT5G48310*), a homologue gene of *CsaV3_6G009810* in *Arabidopsis*, has been shown to activate abiotic stress-related responses such as salt and drought in *Arabidopsis* by encoding an unknown protein that is involved in ABA-dependent signal transduction pathways [42]. In this study, there were significant changes in the expression level of *CsaV3_6G009810* in all the highly salt-tolerant and -sensitive cucumber accessions (Figure 9e).

In fact, the change trend of gene expression in different accessions may be variable. We found that the changes in the expression of five genes in these accessions were diverse, and the expression of the same gene was up-regulated in some of the highly salt-tolerant or -sensitive accessions, while down-regulated in others. For four highly salt-tolerant accessions, the expression of *CsaV3_2G035120* was up-regulated in two accessions, while down-regulated in one accession; the expression of *CsaV3_3G023710* was up-regulated in two accessions, while down-regulated in one accession; the expression of *CsaV3_6G009810* was up-regulated in one accession, while down-regulated in two accessions. For four highly salt-sensitive accessions, the expression of *CsaV3_2G035120* was significantly up-regulated by salt stress in three accessions, while significantly down-regulated in one accession; the expression of *CsaV3_4G033150* was up-regulated in two accessions, while down-regulated in two accessions; the expression of *CsaV3_5G023530* was up-regulated in one accession, while down-regulated in two accessions; the expression of *CsaV3_6G009810* was up-regulated in three accessions, while down-regulated in one accession. In addition, we also found that the expression of *CsaV3_5G023530* was down-regulated in two cucumber accessions under salt stress, while its homologous gene in *Arabidopsis* was significantly up-regulated. We think that the possible reason for this result is not only that the gene expression is variable, but also that the regulation mechanism of this gene in cucumber is different from that in *Arabidopsis*. In any case, the up-regulation or down-regulation of gene expression is strong evidence that these genes respond to salt stress in cucumber.

To sum up, *CsaV3_2G035120*, *CsaV3_3G023710*, *CsaV3_4G033150*, *CsaV3_5G023530,* and *CsaV3_6G009810* are predicted to be involved in the salt stress response in cucumber. The next research will focus on the functional validation of these candidate genes via overexpression and CRISPR in cucumber. This work could provide some references for elucidating the mechanism of cucumber salt stress response and breeding salt-tolerant cucumber cultivars. In addition, the highly salt-tolerant and highly salt-sensitive accessions that were identified in this study can be used as experimental materials for the follow-up gene cloning and salt-tolerant cucumber cultivars breeding, such as selecting accessions to construct RILs populations for QTL mapping.

## 4. Materials and Methods

### 4.1. Plant Materials

The plant materials with a total of 220 accessions that were used in this study are from the cucumber research group of the Institute of Vegetables and Flowers, Chinese Academy of Agricultural Sciences, Beijing, China. The code and ecotype information of 220 accessions can be found in Table S1.

### 4.2. Evaluation of Salt Tolerance Ability and Data Analysis

The salt treatment experiments were carried out in two different environments, including Shouguang (36°51′ N, 118°50′ E) in June 2020 and Beijing (39°9′ N, 116°3′ E) in May 2021. A total of three replicates were set for each experiment, each of which had 5 plants. The cucumbers were cultivated in 54 cm × 28 cm × 9 cm soil pots which had sufficient nursery substrate, with trays for water collection at the bottom. The nursery substrate that was used in each experiment was the same, ideal for cucumber growth and did not affect the salt stress responses. The average ambient temperature in the greenhouse during the experiments was 26.65 ℃ (Shouguang) and 25.05 ℃ (Beijing), respectively. At 14 days old, the seedlings were exposed to salt stress by adding 2 L of 200 mmol·L^−1^ NaCl into the trays regularly to ensure that each seedling was saturated with the same amount of salt solution. The first true leaf and cotyledon showed different degrees of chlorosis and wilting after 7 days of salt treatment. The salt treatment and the calculation of the salt injury index were carried as described by Liu, et al. [7]. The salt injury index was calculated as below: salt injury index = S × ∑(s × *n*)/*N*. Where “S” represents the highest salt injury rating scale, “s” represents the salt injury grade, “*n*” represents the number of plants in the salt injury grade, and “*N*” represents the total number of plants. For each of the cucumber seedlings that were grown under 200 mmol·L^−1^ NaCl, the phenotypic data were collected for the degree of salt injury. The salt symptoms within the 220 cucumber accessions were divided into six scales. WPS Office v.11.1 (Kingsoft, Beijing, China) was used to calculate salt injury index.

Variance analysis and cluster analysis of the phenotypic data were implemented using SPSS v.19.0 (International Business Machines Corporation, Armonk, NY, USA). The cluster analysis used the Ward method and the Euclidean distance method for measurement.

### 4.3. Genome-Wide Association Study (GWAS) and Linkage-Disequilibrium (LD) Analysis

The re-sequenced genome data and SNPs of these 220 core accessions are available in the NCBI Database (PRJNA171718 and PRJNA831637). A general linear model (GLM) was used for the association tests, with an estimated relatedness matrix as a covariate [43]. GWAS was conducted, and the genome-wide lowest *p*-value was recorded. The 5% lowest tail was taken from the 200 recorded minimal *p*-values as the threshold for genome-wide significance.

The software Plink [44] was used to calculate the LD coefficient (*r*^2^) between the pairwise high-quality SNPs and the results were used to estimate LD decay [16]. Manhattan plots and the LD heatmaps were drawn by qqman package in R environment [45].

### 4.4. Identification of QTLs and Candidate Gene Analysis

The regions of the significant trait-associated SNPs were identified as candidate regions of QTLs by repeated loci of two sets of the salt injury index. The cucumber genome database website CuGenDB (http://cucurbitgenomics.org/ (accessed on 2 August 2021)) was used to retrieve candidate resistance genes that were located within a 100-kb interval, from 50 kb upstream and downstream of each peak SNPs from the candidate regions [46]. Gene function was analyzed and predicted by BLAST in the CuGenDB, Swiss-Prot (https://www.uniprot.org/ (accessed on 2 August 2021)), TAIR (https://www.arabidopsis.org/index.jsp (accessed on 2 August 2021)) and Gene Ontology databases (http://amigo.geneontology.org/amigo/landing (accessed on 2 August 2021)) [47]. Furthermore, highly salt-tolerant and highly salt-sensitive core accessions that were obtained by cluster analysis were used as materials, and the genes were analyzed by combination of germplasm haplotype analysis and qRT-PCR to determine the candidate genes that were related to salt tolerance.

### 4.5. RNA Extraction and qRT-PCR Verification

Using four highly salt-tolerant accessions (R45, R63, R78, and CG104) and four highly salt-sensitive accessions (R59, R132, CG13, and CG14) as experimental materials, a total of 4 time points were set, namely 0 h, 10 h, 48 h, and 96 h after salt treatment. There were three replicates for each time point. The first true leaf of each seedling was harvested and immediately put into liquid nitrogen, and stored at −80 ℃. The TaKaRa MiniBEST Universal RNA Extraction Kit (TakaRa, Kusatsu, Japan) was used to extract the total RNA of each sample that was collected at the different time points. Reverse transcription of the extracted RNA into cDNA was performed with the UEIris II RT-PCR System (Biodee, Beijing, China). According to the CDS sequence of the candidate gene on the reference genome, primer 6.0 software was used to design the primers (Table S6). qRT-PCR was performed with ChamQ Universal SYBR qPCR Master Mix (Vazyme, Nanjing, China), using the CFX96 Real-Time System (Bio-Rad, Hercules, CA, USA). Analysis of the relative expression data of the candidate genes was performed using the 2^−ΔΔCt^ method [48].

## 5. Conclusions

We reported 58 highly salt-tolerant accessions and 20 highly salt-sensitive accessions from 220 cucumber core accessions. A total of seven loci, namely *gST2.1*, *gST3.1*, *gST3.2*, *gST4.1*, *gST4.2*, *gST5.1,* and *gST6.1*, that regulate salt tolerance in cucumber seedlings, were repeatedly detected. Furthermore, five candidate genes (*CsaV3_2G035120*, *CsaV3_3G023710*, *CsaV3_4G033150*, *CsaV3_5G023530*, and *CsaV3_6G009810*) within these loci that are involved in the salt stress response were predicted through gene analysis, haplotype, and qRT-PCR analyses. This study not only provides a reference for the subsequent excavation of candidate genes and the molecular mechanism of salt tolerance in cucumber, but also provides a theoretical basis for cultivating new high-yield and high-quality cucumber varieties with highly salt-tolerant, which will accelerate the cultivation of salt-tolerant cucumber varieties.

## Figures and Tables

**Table 1:** The salt injury indexes of the germplasm in two years.

Treatment	Max. Score	Min. Score	Mean	STD	Skewness	Kurtosis	C.V	Normal Distribution Test (*p* Value)
Salt_2020	63.00	3.00	31.18	12.89	−0.05	−0.25	41.35%	0.83
Salt_2021	69.30	6.95	28.80	13.78	0.77	0.22	47.84%	0.06

## Data Availability

Not applicable.

## Supplementary Materials

The following supporting information can be downloaded at: https://www.mdpi.com/article/10.3390/ijms23158260/s1.
