# Supplementary material for: A Large-Scale Genomic Association Analysis Identifies the Candidate Genes Regulating Salt Tolerance in Cucumber (Cucumis sativus L.) Seedlings
Source: Int J Mol Sci. 2022 Jul 27;23(15):8260. doi: 10.3390/ijms23158260 (PMC9332819; doi:10.3390/ijms23158260)
Supplement: Supplementary file 1 [file ijms-23-08260-s001.zip › Supplementary Figures.pdf]

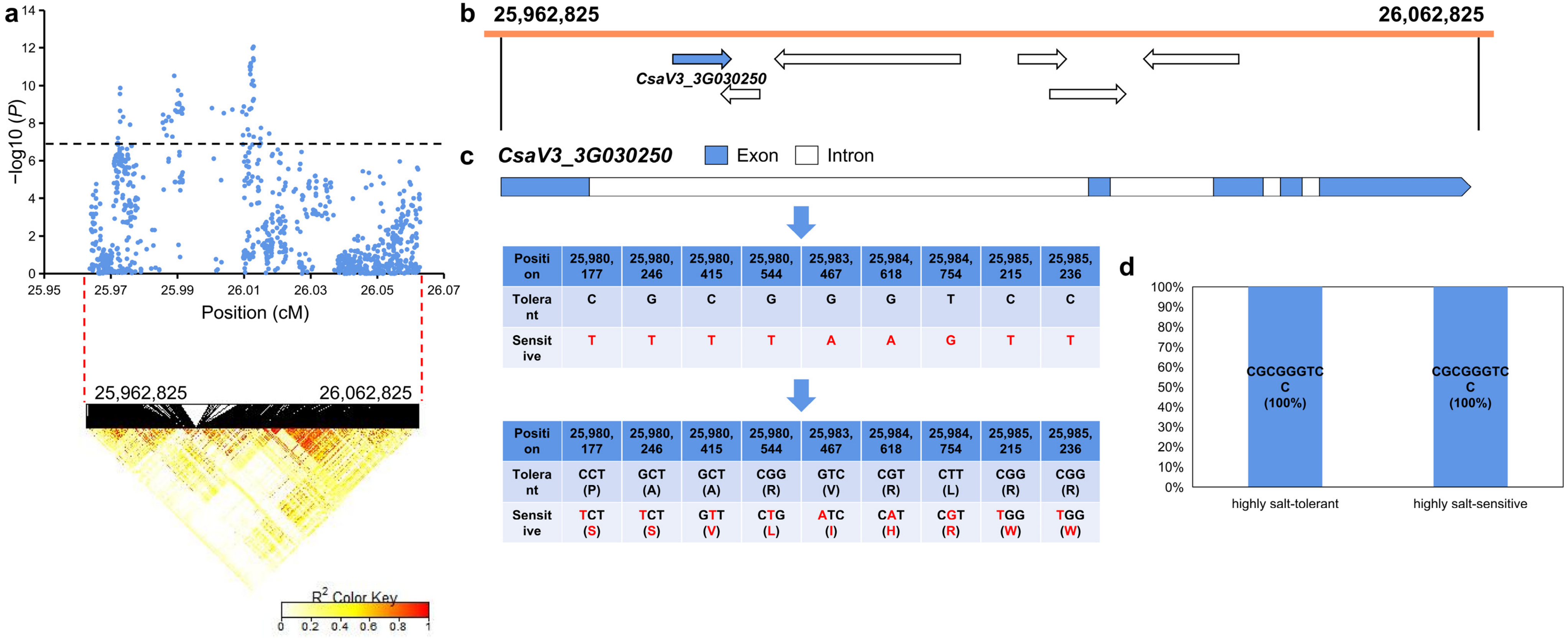

Figure S1. Identification of the candidate gene related to salt tolerance for the locus *gST3.2*.

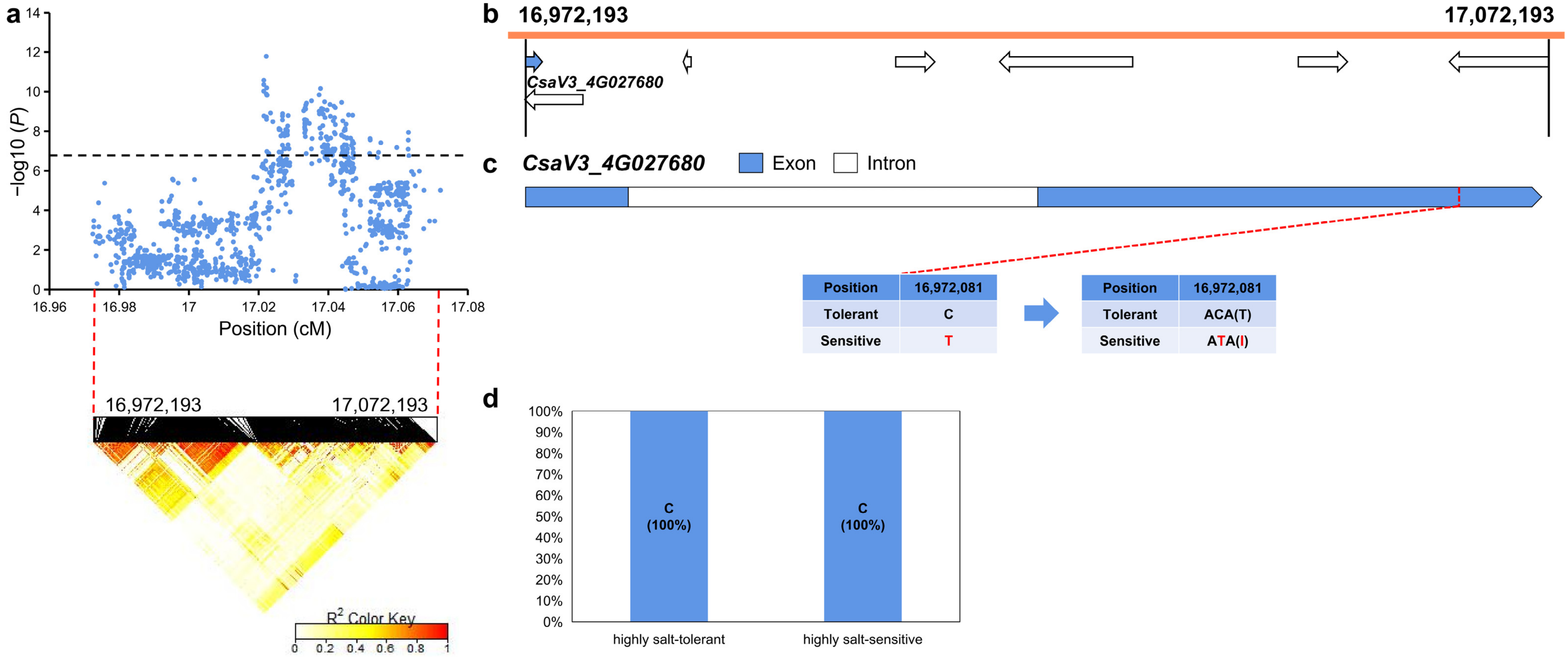

Figure S2. Identification of the candidate gene related to salt tolerance for the locus *gST4.1*.
