# Supplementary material for: A Large-Scale Genomic Association Analysis Identifies the Candidate Genes Regulating Salt Tolerance in Cucumber (Cucumis sativus L.) Seedlings
Source: Int J Mol Sci. 2022 Jul 27;23(15):8260. doi: 10.3390/ijms23158260 (PMC9332819; doi:10.3390/ijms23158260)
Supplement: Supplementary file 1 [file ijms-23-08260-s001.zip › Supplementary Tables.pdf]

Table S1 Ecotype information on plant materials for salt tolerance research

Code	Ecotype
R1	European
R2	North China
R3	South China
R4	North China-European hybrid
R5	North China-European hybrid
R6	North China-European hybrid
R7	North China-European hybrid
R13	North China
R14	North China
R15	North China
R16	North China
R17	North China-European hybrid
R18	North China-European hybrid
R19	North China
R20	North China
R21	North China
R22	North China
R23	North China
R24	North China
R25	North China
R26	North China
R27	North China-European hybrid
R28	North China
R29	North China
R30	North China
R31	North China-European hybrid
R32	North China-European hybrid
R33	North China-European hybrid
R34	North China
R35	North China
R36	North China-European hybrid
R37	North China-European hybrid
R38	North China-European hybrid
R39	North China-European hybrid
R40	North China
R41	North China-European hybrid
R42	North China-European hybrid
R43	North China-European hybrid
R44	North China-European hybrid
R45	North China
R47	European
R48	European
R49	North China
R50	North China
R51	North China
R52	North China
R53	North China
R54	North China

R55	North China
R56	North China
R57	American processing
R58	American processing
R59	European-Asia
R60	North China
R62	South China
R63	South China
R64	South China
R65	American slicing
R67	North China
R68	North China
R69	South China
R70	South China
R71	North China
R72	North China
R73	North China
R74	North China
R75	European
R76	European
R77	European
R78	European
R80	European
R81	European
R82	European
R83	European
R84	European
R85	European
R86	European
R87	European
R88	European
R89	South China
R90	South China
R91	South China
R92	South China
R93	South China
R94	South China
R95	South China
R96	South China
R97	South China
R98	South China
R99	South China
R100	South China
R101	North China
R102	North China
R103	North China
R104	North China
R105	North China
R106	North China
R107	North China

R108	North China
R109	North China
R110	North China
R111	North China
R112	North China
R113	North China
R114	North China
R115	North China
R116	North China
R117	North China
R118	North China
R119	North China
R120	Japan
R121	Japan
R122	Japan
R123	Japan
R124	Japan
R125	American processing
R126	American slicing
R127	American slicing
R128	American processing
R129	American processing
R130	European
R131	European
R132	India
R133	South China
R134	American processing
R135	American processing
R136	American processing
R137	American processing
R138	American processing
R139	American slicing
R140	American slicing
R141	Japan
R142	South China
R143	South China
R148	American processing
R150	North China
R151	North China
R152	North China
R153	North China
R154	North China
R155	North China
R157	North China
R159	North China
R160	North China
R161	North China
R162	North China
R163	North China
R164	North China

R165	North China
R166	South China
R167	South China
R168	North China
R169	North China
R170	North China
R171	North China
R172	North China
R174	North China
R175	North China
R176	North China
R177	South China
CG1	European
CG2	India
CG3	Japan
CG4	Japan
CG5	European
CG9	American processing
CG10	European
CG11	European
CG12	India
CG13	European
CG14	India
CG15	India
CG16	India
CG17	India
CG19	India
CG21	India
CG23	India
CG25	North China
CG28	Japan
CG29	Japan
CG31	Japan
CG32	European
CG35	European
CG36	American processing
CG40	European
CG43	American processing
CG44	American processing
CG45	American processing
CG49	India
CG50	India
CG51	India
CG59	South China
CG66	Xishuangbanna
CG72	Xishuangbanna
CG77	Xishuangbanna
CG78	Xishuangbanna
CG84	Xishuangbanna
CG85	Xishuangbanna

CG87	India
CG88	India
CG89	North China
CG91	North China
CG92	South China
CG94	North China
CG96	North China
CG98	South China
CG99	South China
CG100	North China
CG104	Japan
CG106	South China
CG107	North China
CG108	North China
CG109	European
CG110	European
CG112	European
CG113	European
CG114	European
CG117	India
CG118	India
CG120	South China

Table S2 Phenotypic data of 220 cucumber accessions

Code	June 2020	May 2021
R1	44.9	48.0
R2	30.0	20.4
R3	34.2	28.5
R4	37.2	30.6
R5	23.5	13.5
R6	31.5	10.4
R7	34.2	26.1
R13	31.7	22.2
R14	35.1	29.4
R15	45.0	39.4
R16	42.0	23.5
R17	29.4	12.9
R18	13.1	19.0
R19	32.4	16.8
R20	25.8	13.8
R21	44.4	24.6
R22	39.0	17.4
R23	38.7	25.8
R24	33.1	20.3
R25	39.4	26.6
R26	34.7	15.8
R27	49.5	37.5
R28	36.6	26.4
R29	37.4	40.5
R30	27.0	21.4
R31	37.8	26.6
R32	31.8	27.9
R33	21.0	15.6
R34	61.2	22.6
R35	30.0	33.6
R36	33.0	27.2
R37	31.1	23.4
R38	32.9	36.6
R39	14.9	10.2
R40	36.0	49.5
R41	32.6	31.8
R42	28.2	21.0
R43	42.0	37.8
R44	28.8	35.4
R45	5.6	7.0
R47	24.0	15.6
R48	21.6	19.4
R49	8.1	10.4
R50	23.2	24.2
R51	32.3	32.9
R52	8.1	7.2

R53	19.6	12.6
R54	27.0	23.4
R55	29.3	27.0
R56	21.8	9.6
R57	39.2	40.8
R58	32.9	18.0
R59	55.8	52.2
R60	48.6	40.2
R62	3.6	11.7
R63	7.2	11.7
R64	21.0	27.0
R65	21.6	17.7
R67	27.6	27.6
R68	38.4	31.8
R69	48.6	32.6
R70	43.8	38.7
R71	33.9	29.7
R72	35.1	21.0
R73	45.0	54.0
R74	23.4	51.0
R75	33.6	22.8
R76	13.8	16.2
R77	16.8	11.6
R78	12.0	12.9
R80	21.6	45.0
R81	20.4	16.5
R82	24.6	14.3
R83	11.0	20.4
R84	22.2	16.2
R85	15.8	21.0
R86	22.5	9.3
R87	22.5	NA
R88	26.1	NA
R89	37.2	36.0
R90	22.1	12.0
R91	35.5	37.8
R92	26.7	19.8
R93	29.8	15.0
R94	16.2	22.2
R95	36.0	29.4
R96	43.2	37.8
R97	18.0	8.3
R98	27.0	14.4
R99	24.8	14.0
R100	27.0	26.2
R101	26.4	16.2
R102	34.8	38.0
R103	41.4	28.2

R104	3.0	12.6
R105	29.7	18.2
R106	16.5	21.6
R107	37.8	34.2
R108	27.0	23.4
R109	30.6	28.2
R110	34.2	41.4
R111	25.2	10.4
R112	15.8	16.8
R113	36.0	15.8
R114	36.6	13.5
R115	27.0	11.1
R116	10.5	9.0
R117	43.2	46.4
R118	33.8	28.8
R119	12.6	18.0
R120	7.2	13.2
R121	17.4	15.3
R122	25.2	23.8
R123	42.0	38.4
R124	5.0	10.8
R125	15.3	38.6
R126	20.4	28.2
R127	44.1	37.8
R128	27.6	34.2
R129	22.2	46.5
R130	7.8	23.8
R131	55.0	46.2
R132	57.0	69.0
R133	45.0	55.2
R134	36.6	35.1
R135	36.3	27.9
R136	25.5	52.2
R137	23.1	21.6
R138	5.6	14.8
R139	7.1	57.9
R140	27.6	69.3
R141	27.0	32.7
R142	32.4	26.7
R143	31.0	36.6
R148	43.2	41.1
R150	38.7	23.4
R151	25.8	36.6
R152	36.8	42.3
R153	22.5	20.6
R154	45.9	21.6
R155	35.4	30.0
R157	30.0	22.4

R159	31.5	16.5
R160	35.4	36.6
R161	34.8	31.5
R162	30.6	25.5
R163	11.3	15.0
R164	23.4	27.8
R165	21.8	18.4
R166	43.8	22.2
R167	33.0	45.0
R168	40.0	35.1
R169	51.0	37.0
R170	46.8	37.8
R171	35.1	25.8
R172	24.8	21.0
R174	5.4	8.6
R175	39.6	22.8
R176	47.4	36.0
R177	40.5	38.7
CG1	7.4	14.1
CG2	42.0	16.2
CG3	21.2	17.3
CG4	23.4	18.3
CG5	18.0	20.3
CG9	29.4	22.2
CG10	26	20.4
CG11	34.8	25.8
CG12	51	49.2
CG13	63	63
CG14	56.25	60
CG15	49.5	53.7
CG16	36	24.9
CG17	40.2	30.6
CG19	47.4	48.6
CG21	49.5	51
CG23	34.2	27.7
CG25	19.8	51.6
CG28	22.5	34.5
CG29	4.8	28.2
CG31	47.25	41.9
CG32	42.6	29.7
CG35	32.25	34.2
CG36	34.5	25.8
CG40	30.6	27.6
CG43	40.05	30.2
CG44	33	28.5
CG45	39	39.9
CG49	45.3	39
CG50	61.8	55

CG51	27	22.35
CG59	33.75	44.4
CG66	12	NA
CG72	45	66.6
CG77	63	66.15
CG78	12.6	31.8
CG84	40.275	67.8
CG85	40.5	11.7
CG87	52.2	52.5
CG88	40.5	57
CG89	43.5	18.75
CG91	10.2	25.2
CG92	50.7	41.7
CG94	25.2	30
CG96	40.5	50.4
CG98	42.3	36.9
CG99	31.05	16.2
CG100	16.5	9
CG104	4.95	9
CG106	21.6	33.6
CG107	42.6	27
CG108	17.25	21
CG109	40.5	33.6
CG110	33.6	30
CG112	51	36.9
CG113	54	42
CG114	33.4	28.2
CG117	34.2	20.6
CG118	54	51.75
CG120	39.6	28.35

Table S3 Group of accessions for salt tolerance in seedling stage

Salt tolerancce Groups	Groups	Code
highly salt-tolerant group	I	R45, CG104, R174, R62, R52, R104, R124, R49, R63, R116, R138, R120, CG1, CG66, R78, R39, CG100, R97, R163, R77, R76, R119, R56, R83, R130, R86, R18, R53, R112, R121, CG29, R90, CG91, R111, R33, R85, R81, R5, R115, R106, CG5, CG108, R84, R94, CG3, R99, R82, R65, R20, R47, R165, R48, R98, CG4, R6, R17, R101, R153
salt-tolerant group	II	CG78, R137, R93, R87, R172, CG10, R92, CG99, R50, R105, R64, R159, R30, R126, R122, R19, R42, CG51, R114, R2, R26, R54, R108, R58, R164, CG9, R113, R88, CG85, R157, R100, R24, R13, R125, R37, CG117, R67, CG94, CG106, R72, R162, R55, R22, R75, CG28
intermediate salt-tolerant group	III	CG2, CG40, R109, R142, R141, R32, R36, CG36, R7, CG11, CG16, R171, CG44, CG114, R128, CG23, R150, CG89, R151, R175, R118, R3, R28, R71, CG110, R35, R44, R135, R41, R31, R14, R23, R139, R51, R95, R155, R16, R25, R166, R161, CG35, R80, R154, R143, R4, CG120, R129, R21, R38, R103, CG107, R68, CG43, CG17, CG25, R134, R107, R160, CG32, R102, R89, R91, CG109, R74, R168, R110
salt-sensitive group	IV	R136, R29, R167, CG59, CG45, R152, R177, CG98, R43, R57, R123, R96, R69, R127, R70, R176, R34, R148, CG49, R15, R170, R40, R27, CG112, R169, R60, CG31, R117, CG96, CG92, R1
highly salt-sensitive group	V	CG19, CG113, R140, CG88, R73, R133, CG12, CG21, R131, CG15, CG87, CG118, R59, CG84, CG72, CG14, CG50, R132, CG13, CG77

Table S4 Signal locus and genes information detected by GWAS analysis

GWAS signal region	Gene name	physical location
gST2.1	*CsaV3_2G03504(*	23407980-23412070
	CsaV3_2G03505(	23415651-23419542
	CsaV3_2G03506(	23419391-23419631
	CsaV3_2G03507(	23428043-23434112
	CsaV3_2G03508(	23433845-23434050
	CsaV3_2G03509(	23458082-23459247
	CsaV3_2G03510(	23475335-23479035
	CsaV3_2G03511(	23480766-23489469
	CsaV3_2G03512(	23490932-23493700
	CsaV3_2G03513(	23501857-23505429
	CsaV3_2G03514(	23505456-23506957
gST3.1	*CsaV3_3G02370(*	21489794-21514458
	CsaV3_3G02371(	21522682-21532658
	CsaV3_3G02372(	21540330-21542136
	CsaV3_3G02373(	21549957-21553175
	CsaV3_3G02374(	21554667-21555910
	CsaV3_3G02375(	21569210-21577666
gST3.2	*CsaV3_3G03024(*	25959150-25964239
	CsaV3_3G03025(	25980074-25985538
	CsaV3_3G03026(	25985730-25989154
	CsaV3_3G03027(	25991251-26009604
	CsaV3_3G03028(	26015518-26019865
	CsaV3_3G03029(	26018818-26025938
	CsaV3_3G03030(	26029082-26038086
gST4.1	*CsaV3_4G02768(*	16971087-16972740
	CsaV3_4G02769(	16971478-16977878
	CsaV3_4G02770(	16988121-16988421
	CsaV3_4G02771(	17008408-17011665
	CsaV3_4G02772(	17019206-17031571
	CsaV3_4G02773(	17047856-17052059
	CsaV3_4G02774(	17063296-17073446
gST4.2	*CsaV3_4G03313(*	23431012-23433256
	CsaV3_4G03314(	23433929-23439249
	CsaV3_4G03315(	23439698-23445777
	CsaV3_4G03316(	23453901-23455766
	CsaV3_4G03317(	23459779-23462177
	CsaV3_4G03318(	23465790-23470303
	CsaV3_4G03319(	23474366-23474623
	CsaV3_4G03320(	23492178-23496591
	CsaV3_4G03321(	23497530-23510992
	CsaV3_4G03322(	23512731-23513007
	CsaV3_4G03323(	23516885-23522184
gST5.1	*CsaV3_5G02350(*	17873647-17874229
	CsaV3_5G02351(	17877347-17877500
	CsaV3_5G02352(	17884509-17885702
	CsaV3_5G02353(	17891353-17909145
	CsaV3_5G02354(	17914991-17921238
	CsaV3_5G02355(	17922316-17923497
	CsaV3_5G02356(	17929552-17930189

	CsaV3_5G02357(	17945993-17946614
gST6.1	*CsaV3_6G00977(*	7918201-7918627
	CsaV3_6G00978(	7923874-7924730
	CsaV3_6G00979(	7944527-7951003
	CsaV3_6G00980(	7962016-7971218
	CsaV3_6G00981(	7971665-7978991
	CsaV3_6G00982(	7980131-7980390
	CsaV3_6G00983(	7984667-7987751
	CsaV3_6G00984(	7994027-8000088
	CsaV3_6G00985(	8001447-8004575
	CsaV3_6G00986(	8008859-8009051
	CsaV3_6G00987(	8009088-8009436
	CsaV3_6G00988(	8011748-8019379

Table S5 Candidate genes annotation of GWAS Analysis.

Gene name	Gene annotation	Homologous genes of *Arabidopsis thaliana*	Functions annotation
CsaV3_2G035120	Isocitrate lyase	*AT3G21720*	Encodes a glyoxylate cycle enzyme isocitrate lyase (ICL) involved in salt tolerance.
CsaV3_3G023710	Ankyrin repeat family protein	*At3g12360*	Involved in salt stress tolerance; May act through abscisic acid (ABA) signaling pathways and promote reactive oxygen species (ROS) production.
CsaV3_4G033150	Phosphoenolpyruvate carboxylase protein	*AT1G53310*	Encodes one of four Arabidopsis phosphoenolpyruvate carboxylase proteins; Plays an important role in carbon and nitrogen metabolism.
CsaV3_5G023530	Phd finger protein, putative	*AT1G77800*	PHD finger family protein;(source:Araport11)
CsaV3_6G009810	Pesticidal crystal cry8Ba protein	*AT5G48310*	Responsiveness to abscisic acid (ABA), salt and drought 1; Protein of unknown function that may be involved in stress response.

Table S6 Primers for the real time fluorescence quantitative PCR

Gene name	Forward primer	Reverse primer
CsaV3_2G035120	GCTGCCTCCTTCTCTGTTCCTT	GCCGTCGTGTTAGCTTGAACCT
CsaV3_3G023710	AAGAGGCACGGGTCTGAAATGG	GCGTTGGATCAATGTCAAGAAGCA
CsaV3_4G033150	GCTTTGTACGACAAGCTCTTAG	GGTAGTTTGGATCACGAATTCG
CsaV3_5G023530	AACGCAAAGTAATTCCTTGGAC	CTTATTGAAGAATCGTGGCCAG
CsaV3_6G009810	CGCTTTGCCTCCATTGAAATTCCAT	CGTAATCTCCATCGTCGTCGTCATC
Ubiquitin	CACCAAGCCCAAGAAGATC	TAAACCTAATCACCACCAGC
